# Patients with high nuclear grade pT1-ccRCC are more suitable for radical nephrectomy than partial nephrectomy: a multicenter retrospective study using propensity score

**DOI:** 10.1186/s12957-024-03302-y

**Published:** 2024-01-23

**Authors:** Haozhe Xu, Zhuo Xing, Kai Ai, Jie Wang, Zhengtong Lv, Haitao Deng, Ke Li, Yang Wang, Yuan Li

**Affiliations:** 1 Department of Urology, The Second Xiangya Hospital, Central South University, Changsha, 410011 Hunan China; 2 Department of Urology, Xiangya Hospital, Central South University, Changsha, Hunan China; 3 Department of Oncology, Hunan Cancer Hospital, Changsha, Hunan China; 4 Xiangya School of Medicine, Central South University, Changsha, Hunan China; 5 Department of Urology, Beijing Hospital, National Center of Gerontology, Institute of Geriatric Medicine, Chinese Academy of Medical Sciences, Beijing, China; 6 Department of Pathology, The Second Xiangya Hospital, Central South University, Changsha, 410011 Hunan China

**Keywords:** Clear cell renal cell carcinoma, Nuclear grade, Overlap weighting, Partial nephrectomy

## Abstract

**Background:**

Partial nephrectomy (PN) is usually recommended for T1 stage clear cell renal cell carcinoma (ccRCC) regardless of the nuclear grades. However, the question remains unresolved as to whether PN is non-inferior to RN in patients with T1-ccRCC at higher risk of recurrence. In fact, we found that patients with high nuclear grades treated with PN had poorer prognosis compared with those treated with radical nephrectomy (RN). Therefore, this study was designed to evaluate the associations of PN and RN in the four nuclear grade subsets with oncologic outcomes.

**Methods:**

A retrospective study was conducted in three Chinese urological centers that included 1,714 patients who underwent PN or RN for sporadic, unilateral, pT1, N0, and M0 ccRCC without positive surgical margins and neoadjuvant therapy between 2010 and 2019. Associations of nephrectomy type with local ipsilateral recurrence, distant metastases, and all-cause mortality (ACM) were evaluated using the Kaplan–Meier method and multivariable Cox proportional hazards regression models after overlap weighting (OW).

**Results:**

A total of 1675 patients entered the OW cohort. After OW, in comparison to PN, RN associated with a reduced risk of local ipsilateral recurrence in the G2 subset (HR = 0.148, 95% CI 0.046–0.474; *p* < 0.05), G3 subset (HR = 0.097, 95% CI 0.021–0.455; *p* < 0.05), and G4 subset (HR = 0.091, 95% CI 0.011–0.736; *p* < 0.05), and resulting in increased five-year local recurrence-free survival rates of 7.0%, 17.9%, and 36.2%, respectively. An association between RN and a reduced risk of distant metastases in the G4 subset (HR = 0.071, 95% CI 0.016–0.325; *p* < 0.05), with the five-year distant metastases-free survival rate increasing by 33.1% was also observed. No significant difference in ACM between PN and RN was identified.

**Conclusions:**

Our findings substantiate that opting for RN, as opposed to PN, is more advantageous for local recurrence-free survival and distant metastases-free survival in patients with high nuclear grade (especially G4) pT1-ccRCC. We recommend placing a heightened emphasis on enhancing preoperative nuclear grade assessment, as it can significantly influence the choice of surgical plan.

**Trial registration:**

This study was registered at Chinese Clinical Trial Registry (ID: ChiCTR2200063333).

**Supplementary Information:**

The online version contains supplementary material available at 10.1186/s12957-024-03302-y.

## Introduction

Renal cell carcinoma (RCC) stands as the predominant solid lesion affecting the kidney, with clear cell RCC (ccRCC) constituting its most prevalent histological subtype, accounting for approximately 70%–80% of all subtypes [1]. Partial nephrectomy (PN) is endorsed by a majority of global urological guidelines for T1-ccRCC, as it offers not only comparable tumor control but also superior preservation of renal function compared to radical nephrectomy (RN) [2, 3]. Yet, the question remains unresolved as to whether PN is non-inferior to RN in patients with T1-ccRCC at higher risk of recurrence.

Nuclear grade, commonly used to measure ccRCC malignancy, has demonstrated a robust association with the risk of recurrence. Present study suggested that the patients with high nuclear grades treated with PN deserves more attention and might need adjuvant management because of the tendency of recurrence [4–6]. In fact, we found that the patients with high nuclear grades T1-ccRCC treated with PN have poorer prognosis, which is not consistent with similar published researches. This discrepancy led us to discover that prior studies comparing the efficacy of PN and RN did not account for the possible impact of unbalanced nuclear grades. Specifically, the sample size of high nuclear grades (especially G4) was typically much smaller than that of the low grades [7–10]. If the results are not separately analyzed, the large sample size of low nuclear grade group may obscure the true results of high nuclear grade group. It is important to separately reconsider the oncologic outcomes of the two types of surgery in terms of the different nuclear grades.

To discern whether the oncological outcomes of PN are not inferior to RN in patients with high nuclear grade, we employed a large cohort to evaluate and confirm the distinct efficacy of RN and PN in managing pT1-ccRCC with various nuclear grades. Our findings aim to guide surgeons in selecting the most appropriate surgical options for patients with high nuclear grade T1-ccRCC, thereby enhancing the oncological outcomes for these patients.

## Patients and methods

### Patient selection

After Institutional Review Board approval (ID:202,201,007), we identified patients who underwent PN or RN at three urological centers in China for sporadic, unilateral, pT1, N0, and M0 ccRCC without positive surgical margins and neoadjuvant therapy between 2010 and 2019 (including 1,164 males and 550 females). Patients who underwent robot-assisted surgery were not included in this study. All the surgeons possess more than 5 years of experience in performing RN/PN. The clinical tumor (cT) stage and pathological tumor (pT) stage was determined according to the American Joint Committee on Cancer Cancer Staging Manual (8th edition) [11].

### Patient features

The clinical features were sex, age at surgery, smoking status, lumbago, hematuresis, laterality, Eastern Cooperative Oncology Group (ECOG) performance status, age-adjusted Charlson Comorbidity Index (aCCI), baseline cardiovascular disease (defined using the myocardial infarction, congestive heart failure, and peripheral vascular disease components of the Charlson score), body mass index (BMI), preoperative estimated glomerular filtration rate (eGFR, mL/min/1.73 m2), and surgical approach (open versus laparoscopic). The radiographic features were cT-stage, hemorrhage, necrosis, calcification, and cystic formation. Enhanced computed tomography was used to assess the radiographic features. The pathologic features were rereviewed by 2 genitourinary pathologists, included tumor size, pT-stage and WHO/ISUP grade.

### Outcomes

The main outcomes were local ipsilateral recurrence, distant metastases, and all-cause mortality (ACM). Local ipsilateral recurrence did not include the ipsilateral adrenal gland and distant metastases included the contralateral kidney. The follow-up duration was calculated from the date of treatment to the date of the main outcome or the date of the last follow-up.

### Statistical analysis

The patient features were summarized as frequencies and percentages for categorical variables, and medians and interquartile ranges (IQRs) for continuous not normally distributed variables. To compare patient features between two groups, the Wilcoxon rank-sum test was used for not normally distributed variables, and the Fisher exact test and the chi-square test were used for categorical variables.

Due to the limited sample size in G4 subset, we have refrained from utilizing the PS matching. In the four subsets cohort we used overlap weighting (OW) [12] to balance the confounding factors caused by the nonrandomized design of this study. OW is a recently developed balancing weighting scheme which has been demonstrated to optimize the precision of the estimated association between the treatment and outcomes among a large class of propensity score (PS) weighting methods, including inverse probability treatment weight [13]. We used weight (Wi) according to the following formula: Wi = PS for RN and Wi = 1-PS for PN, where PS is the propensity score. The PS representing the probability of treating with RN was estimated using a logistic regression model with RN as the exposure, and all patient features, except nuclear grade, as the covariates (nuclear grade was used as a variable for subgroups) [14].

A total of 1,714 patients met the inclusion and exclusion criteria, whereas 23 patients who underwent RN and 16 patients who underwent PN were missing data for at least one of the patient characteristics studied. Therefore, a total of 1,675 patients formed the cohort for PS analysis, including 774 (46%) RN patients and 901 (54%) PN patients. The outcome differences were assessed between RN and PN in the four nuclear grade subsets. The probabilities of local ipsilateral recurrence-free, distant metastases-free, and overall survival were estimated using the Kaplan–Meier method, and the associations of treatment and outcome were examined using univariable and multivariable Cox proportional hazards regression models. Multivariable Cox regression was performed for variables with *p* < 0.05 on univariable Cox regression and summarized as hazard ratios (HRs) and 95% confidence intervals (CIs). Statistical analyses were performed using SAS version 9.4 and R version 4.2.2. All statistical tests were two-sided with significance set at *p* < 0.05.

## Results

### Patient features

**Fig. 1:**
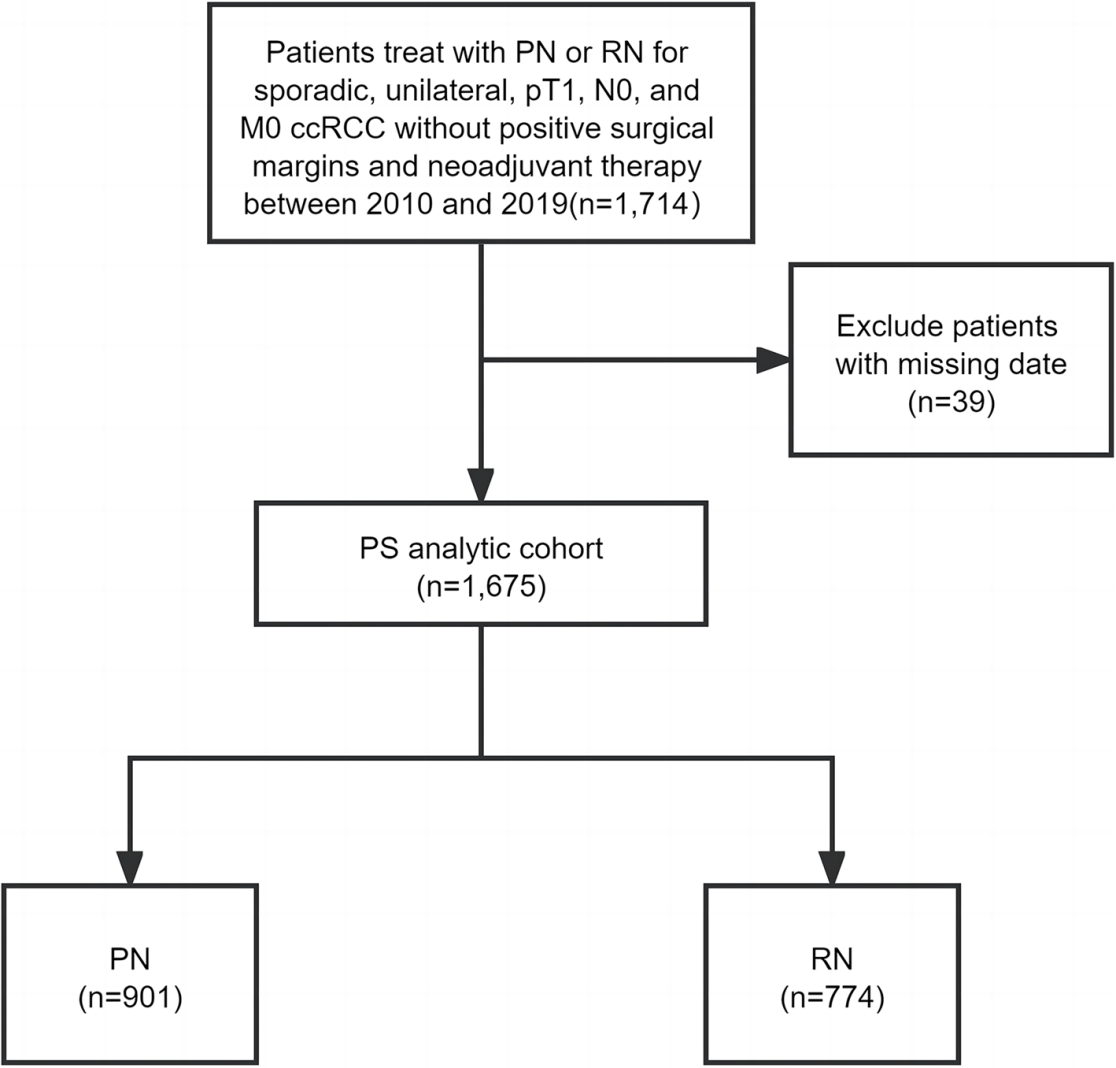
Study flow chart ccRCC = clear cell renal cell carcinoma; RN = radical nephrectomy; PN = partial nephrectomy; PS = propensity score

The patient features are described in Table 1. There were 1,714 patients in the overall cohort, which included 797 (46%) patients who underwent RN and 917 (54%) patients who underwent PN (Fig. 1). The patients who underwent RN were significantly older with a lower preoperative eGFR, more symptoms (lumbago), larger tumors, and higher tumor stages than patients who underwent PN. All features were well balanced between RN and PN groups after PS adjustment (Table 2). The median follow-up time of the 1675 patients included in the PS analysis was 49 (IQR 26, 82) mo. During this time, 48 patients developed local ipsilateral recurrence, 60 developed distant metastases, and 52 died.

**Table 1:** Comparisons of patient features in the overall cohort (*N* = 1,714)

Feature	PN (*N* = 917)	RN (*N* = 797)	*P* value
**Sex**			
**Male**	646 (70)	518 (65)	< 0.05
**Female**	271 (30)	279 (35)	
**Age(years)**	52.6 (45.0–61.0)	54.2 (46.0–62.0)	< 0.05
**Side**			
**Left**	444 (48)	372 (47)	0.471
**Right**	473 (52)	425 (53)	
**Tumor size(cm)**	3.2 (2.5–4.0)	4.5 (3.9–5.2)	< 0.05
**cT Stage**			
**1a**	692(75)	286 (36)	< 0.05
**1b**	225(25)	511 (64)	
**pT Stage**			
**1a**	692 (75)	286 (36)	< 0.05
**1b**	225 (25)	511 (64)	
**Surgical approach**			
**Open**	84 (9)	85(11)	0.297
**Laparoscopic**	833 (91)	712 (89)	
**Lumbago**			
**No**	825(90)	690 (87)	< 0.05
**Yes**	92 (10)	107 (13)	
**Hematuresis**			
**No**	876 (96)	702 (88)	< 0.05
**Yes**	41 (4)	95 (12)	
**Cardiovascular disease**			
**No**	674 (74)	575 (72)	0.529
**Yes**	243 (26)	222 (28)	
**Smoking status**			
**Never**	672 (73)	577 (72)	0.475
**< 10** **years**	64 (7)	45 (6)	
**10–19** **years**	92 (10)	95 (12)	
**20–29** **years**	53 (6)	53 (7)	
**≥ 30** **years**	35 (4)	26 (3)	
**Preoperative eGFR**	80.4 (66.1–97.7)	71.8 (58.2–87.2)	< 0.05
**Preoperative eGFR status**			
**≥ 90**	325 (35)	160 (20)	< 0.05
**60–90**	427 (47)	411 (52)	
**30–60**	153 (17)	211 (26)	
**15–30**	10 (1)	11 (1)	
**≤ 15**	2 (0)	4 (1)	
**Charlson score**	2.0 (2.0–2.0)	2.0 (2.0–2.0)	0.429
**ECOG performance status**			
**0**	480 (52)	367 (46)	0.059
**1**	368 (40)	350 (44)	
**2**	61 (7)	70 (9)	
**3**	2 (0)	2 (0)	
**BMI**	23.6 (21.6–25.6)	23.7 (21.7–26.5)	0.073
**Radiographic evidence of hemorrhage**			
**No**	876 (96)	759 (96)	0.838
**Yes**	40 (4)	33 (4)	
**Radiographic evidence of cysts** **formation**			
**No**	839 (92)	743 (94)	0.080
**Yes**	77 (8)	49 (6)	
**Radiographic evidence of calcification**			
**No**	876 (96)	751 (95)	0.432
**Yes**	40 (4)	41 (5)	
**Radiographic evidence of necrosis**			
**No**	907 (99)	780 (98)	0.319
**Yes**	9 (1)	12 (2)	
**Nuclear grade**			
**1**	266 (29)	176 (22)	< 0.05
**2**	483 (53)	450 (56)	
**3**	136 (15)	133 (17)	
**4**	32 (3)	38 (5)	

Numbers represent median (IQR) or N (%)

*BMI* body mass index, *ECOG* Eastern Cooperative Oncology Group, *eGFR* estimated glomerular filtration rate, *IQR* interquartile range, *PN* partial nephrectomy, *RN* radical nephrectomy

**Table 2:** Comparisons of patient features in the pseudo overall cohort after OW

Feature	PN (*N* = 317)	RN (*N* = 317)	*P* value
Sex			
** Male**	209 (66)	209 (66)	1.000
** Female**	108 (34)	108 (34)	
**Age (years)**	54.0 (46.0–62.0)	54.0 (46.0–62.0)	1.000
Side			
** Left**	151 (48)	151 (48)	1.000
** Right**	166 (52)	166 (52)	
**Tumor size (cm)**	4.0 (3.0–4.7)	4.0 (3.0–4.8)	1.000
**cT Stage**			
** 1a**	180 (57)	180 (57)	1.000
** 1b**	137 (43)	137 (43)	
**pT Stage**			
** 1a**	180 (57)	180 (57)	1.000
** 1b**	137 (43)	137 (43)	
**Surgical approach**			
** Open**	31 (10)	31 (10)	1.000
** Laparoscopic**	285 (90)	285 (90)	
**Lumbago**			
** No**	278 (88)	278 (88)	1.000
** Yes**	38 (12)	38 (12)	
**Hematuresis**			
** No**	299 (94)	299 (94)	1.000
** Yes**	18 (6)	18 (6)	
**Cardiovascular disease**			
** No**	232 (73)	232 (73)	1.000
** Yes**	85 (27)	85 (27)	
**Smoking status**			
** Never**	235 (74)	235 (74)	1.000
** < 10 years**	18 (6)	18 (6)	
** 10–19 years**	32 (10)	32 (10)	
** 20–29 years**	19 (6)	19 (6)	
** ≥ 30 years**	12 (4)	12 (4)	
**Preoperative eGFR**	74.3 (60.6–89.8)	75.9 (60.6–91.7)	1.000
**Preoperative eGFR status**			
** ≥ 90**	78 (25)	85 (27)	0.813
** 60–90**	164 (52)	154 (49)	
** 30–60**	70 (22)	70 (22)	
** 15–30**	3 (1)	6 (2)	
** ≤ 15**	1 (0)	1 (0)	
**aCCI score**	2.0 (2.0–2.0)	2.0 (2.0–2.0)	1.000
**ECOG performance status**			
** 0**	158 (50)	158 (50)	1.000
** 1**	134 (42)	134 (42)	
** 2**	24 (8)	24 (8)	
** 3**	1 (0)	1 (0)	
**BMI**	23.7 (21.9–26.4)	23.7 (21.6–25.8)	1.000
**Radiographic evidence of hemorrhage**			
** No**	302 (95)	302 (95)	1.000
** Yes**	14 (4)	14 (4)	
**Radiographic evidence of cysts degeneration**			
** No**	294 (93)	294 (93)	1.000
** Yes**	23 (7)	23 (7)	
**Radiographic evidence of calcification**			
** No**	308 (97)	308 (97)	1.000
** Yes**	8 (3)	8 (3)	
**Radiographic evidence of necrosis**			
** No**	313 (99)	313 (99)	1.000
** Yes**	4 (1)	4 (1)	
**Nuclear Grade**			
** 1**	75 (24)	87 (27)	0.571
** 2**	177 (56)	163 (51)	
** 3**	55 (17)	53 (17)	
** 4**	10 (3)	13 (4)	

The numbers represent median (IQR) or N (%)

*aCCI* age-adjusted Charlson Comorbidity Index, *OW* overlap weighting, *IQR* interquartile range, *PN* partial nephrectomy, *RN* radical nephrectomy, *BMI* body mass index; *eGFR* estimated glomerular filtration rate

We divided the overall cohort into four subsets, G1–G4, based on the different nuclear grades. As presented in Supplementary Tables 1–4, all patient features were balanced in the subsets after reweighting by OW, except preoperative eGFR in the G1 subset; tumor size in the G2 subset; and tumor size, cT stage, pT stage, and surgical approach in the G3 subset. The remaining imbalanced features were further adjusted by regression in survival analysis.

### Subset analysis

#### Survival analysis of the G1 subset

**Fig. 2:**
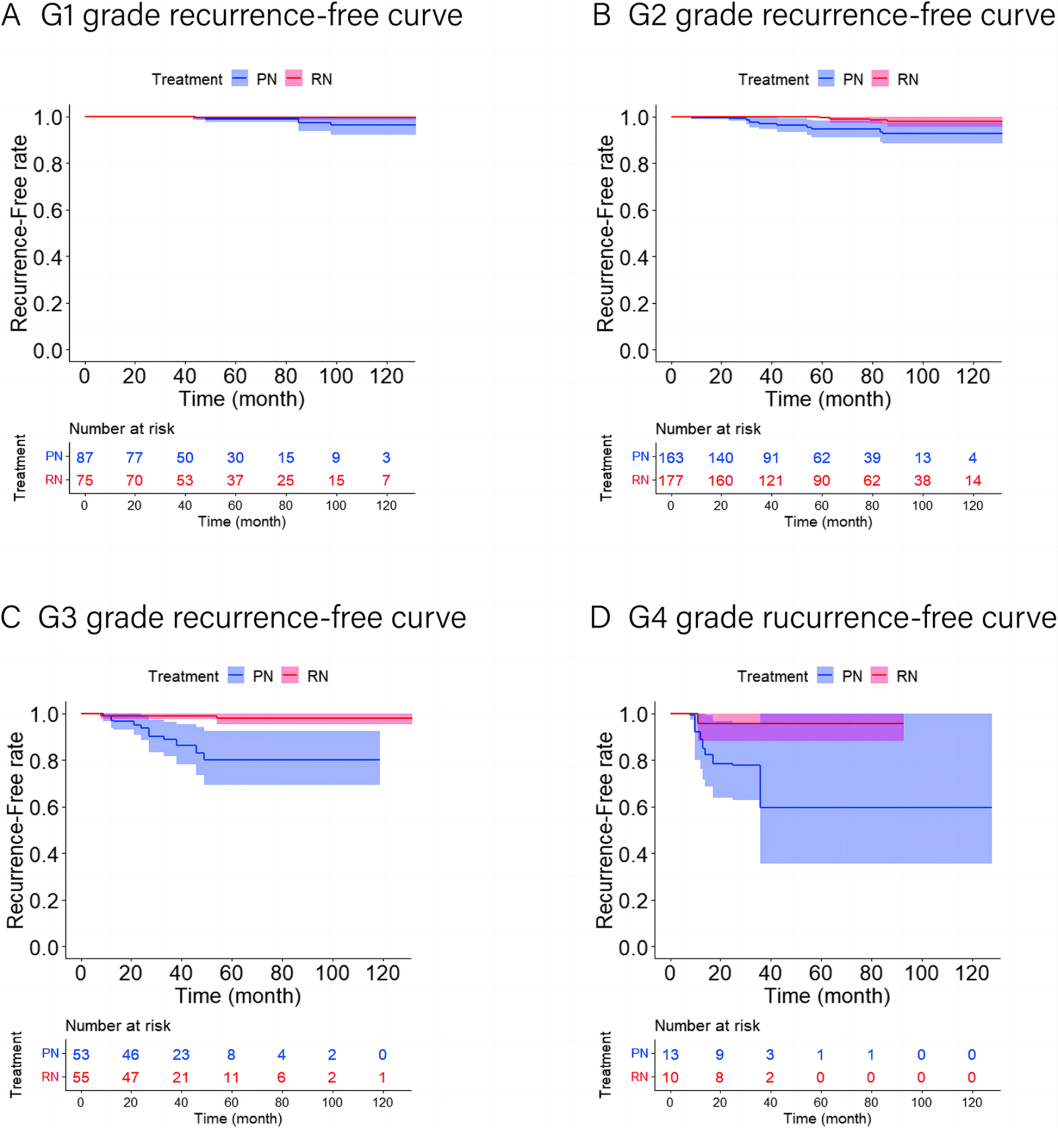
Local recurrence-free curves for RN versus PN after OW. **A** Local recurrence-free curve for RN versus PN in G1 subset after OW (*p* = 0.518). **B** Local recurrence-free curve for RN versus PN in G2 subset after OW (*p* < 0.05). **C** Local recurrence-free curve for RN versus PN in G3 subset after OW (*p* < 0.05). **D** Local recurrence-free curve for RN versus PN in G4 subset after OW (*p* < 0.05). The shaded area denotes the 95% confidence interval for the predicted probability. OW = overlap weighting; PN = partial nephrectomy; RN = radical nephrectomy

**Fig. 3:**
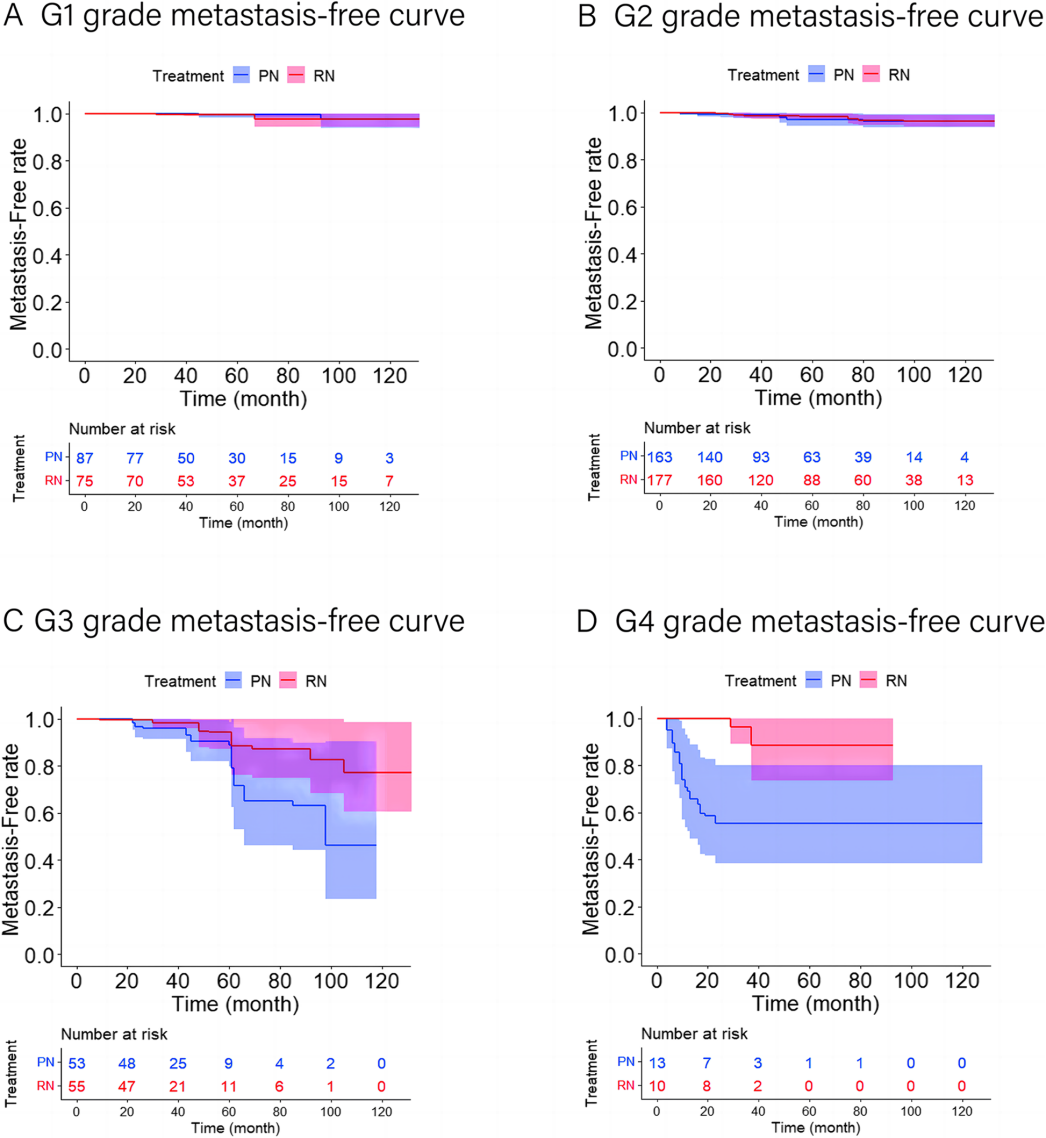
Distant metastases-free curves for RN versus PN after OW. **A** Distant metastases-free curve for RN versus PN in G1 subset after OW (*p* = 0.788). **B** Distant metastases-free curve for RN versus PN in G2 subset after OW (*p* = 0.699). **C** Distant metastases-free curve for RN versus PN in G3 subset after OW (*p* < 0.05). **D** Distant metastases-free curve for RN versus PN in G4 subset after OW (*p* < 0.05). The shaded area denotes the 95% confidence interval for the predicted probability. OW = overlap weighting; PN = partial nephrectomy; RN = radical nephrectomy

**Fig. 4:**
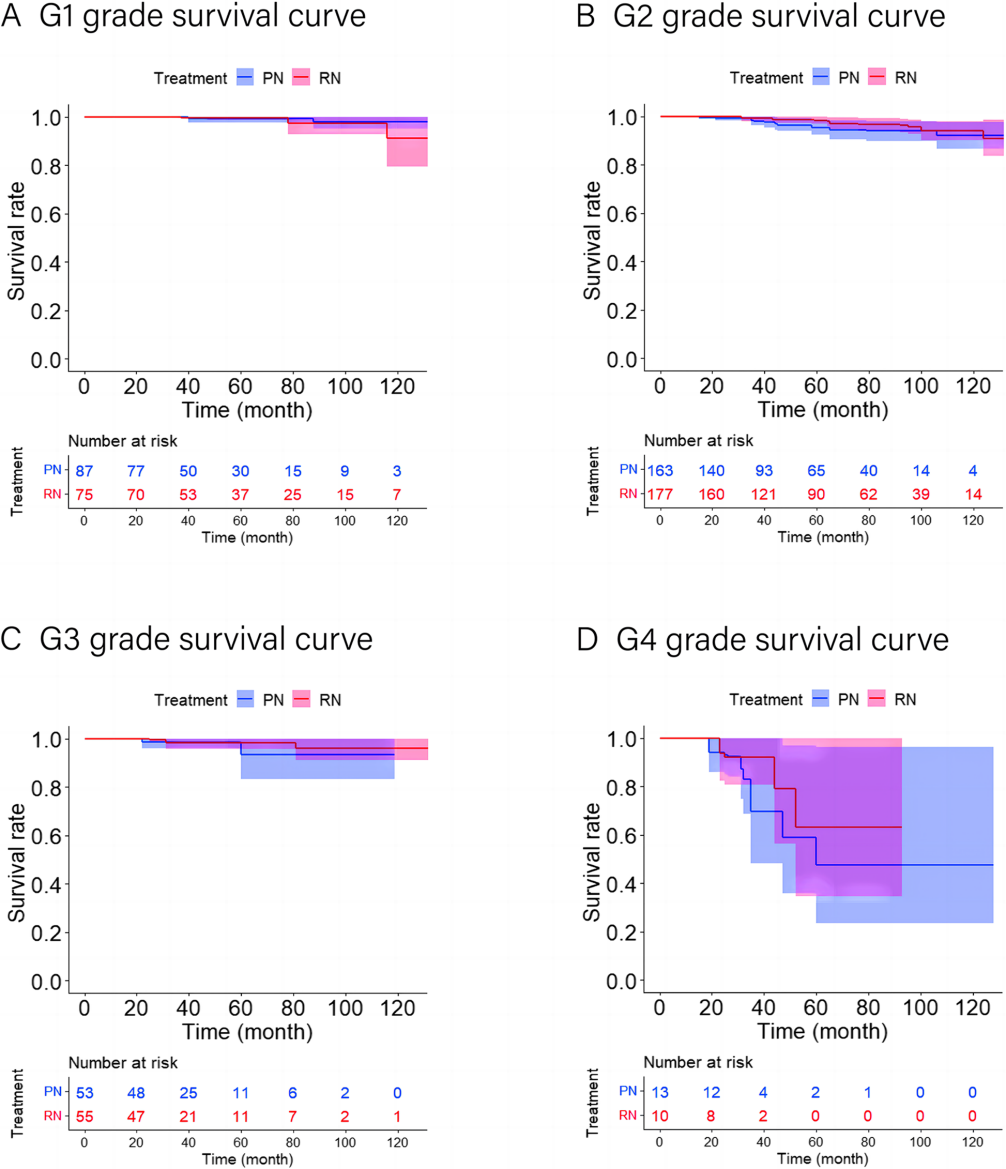
Overall survival curves for RN versus PN after OW. **A** Overall survival curve for RN versus PN in G1 subset after OW (*p* = 0.663). **B** Overall survival curve for RN versus PN in G2 subset after OW (*p* = 0.246). **C** Overall survival curve for RN versus PN in G3 subset after OW (*p* = 0.642). **D** Overall survival curve for RN versus PN in G4 subset after OW (*p* = 0.439). The shaded area denotes the 95% confidence interval for the predicted probability. OW = overlap weighting; PN = partial nephrectomy; RN = radical nephrectomy

The five-year local ipsilateral recurrence-free survival rates, five-year distant metastases-free survival rates, and five-year overall survival rates for patients who underwent RN and PN were 99.5% and 99.0%, 99.5% and 99.5%, and 99.5% and 99.2%, respectively (Figs. 2, 3 and 4). After applying the multivariable Cox proportional hazards model and further adjusting by the preoperative eGFR, no statistical differences in the risk of local ipsilateral recurrence, distant metastases, and ACM between PN and RN were observed (Table 3).

**Table 3:** Associations of nephrectomy type with oncologic outcomes in the four subsets cohort after further adjusting for the imbalanced features

Outcome	HR (95%CI)	*P* value
**Local ipsilateral recurrence**		
** G1 subset**	0.305 (0.029–3.211)	0.323
** G2 subset**	0.148 (0.046–0.474)	< 0.05
** G3 subset**	0.097 (0.021–0.455)	< 0.05
** G4 subset**	0.091 (0.011–0.736)	< 0.05
**Distant metastases**		
** G1 subset**	0.800 (0.167–3.821)	0.780
** G2 subset**	0.750 (0.259–2.174)	0.596
** G3 subset**	0.339 (0.107–1.076)	0.066
** G4 subset**	0.071 (0.016–0.325)	< 0.05
**All-cause mortality**		
** G1 subset**	0.197 (0.018–2.149)	0.183
** G2 subset**	0.619 (0.237–1.62)	0.329
** G3 subset**	0.494 (0.021–11.788)	0.663
** G4 subset**	0.957 (0.275–3.337)	0.945

HR represents the association of RN versus PN with outcome. HR > 1 indicates an increased risk of outcome in patients receiving RN

*CI* confidence interval, *HR* hazard ratio, *PN* partial nephrectomy, *RN* radical nephrectomy

#### Survival analysis of the G2 subset

The five-year local ipsilateral recurrence-free survival rates, five-year distant metastases-free survival rates, and five-year overall survival rates for patients who underwent RN and PN were 99.3% and 92.3%, 98.1% and 94.7%, and 98.2% and 94.8%, respectively (Figs. 2, 3 and 4). After applying the multivariable Cox proportional hazards model and further adjusting by tumor size, RN associated with a significantly reduced risk of local ipsilateral recurrence (HR = 0.148, 95% CI 0.046–0.474; *p* < 0.05) compared with PN. No statistical differences in the risk of distant metastases and ACM between PN and RN were observed (Table 3).

#### Survival analysis of the G3 subset

The five-year local ipsilateral recurrence-free survival rates, five-year distant metastases-free survival rates, and five-year overall survival rates for patients who underwent RN and PN were 98.0% and 80.1%, 94.4% and 88.7%, and 98.2% and 99.3%, respectively (Figs. 2, 3 and 4). After applying the multivariable Cox proportional hazards model and further adjusting by tumor size, cT stage, pT stage, and surgical approach, RN associated with a significantly reduced risk of local ipsilateral recurrence (HR = 0.097, 95% CI 0.021–0.455; *p* < 0.05) compared with PN. No statistical differences in the risk of distant metastases and ACM between PN and RN were observed (Table 3).

#### Survival analysis of the G4 subset

The five-year local ipsilateral recurrence-free survival rates, five-year distant metastases-free survival rates, and five-year overall survival rates for patients who underwent RN and PN were 95.9% and 59.7%, 88.7% and 55.6%, and 63.1% and 47.7%, respectively (Figs. 2, 3 and 4). Compared with PN, RN associated with a significantly reduced risk of local ipsilateral recurrence (HR = 0.091, 95% CI 0.011–0.736; *p* < 0.05) and distant metastases (HR = 0.071, 95% CI 0.016–0.325; *p* < 0.05). No statistical difference in ACM between PN and RN was identified (Table 3).

## Discussion

In subset analysis, we observed that RN was linked to a decreased risk of local recurrence in G2 (HR = 0.148), G3 (HR = 0.097), and G4 (HR = 0.091) subsets, with increased five-year local recurrence-free survival rates of 7.0%, 17.9%, and 36.2%, respectively. Although the oncologic outcomes of PN and RN in the treatment of pT1-ccRCC have been widely studied [1, 7, 8, 15–19], this equivalence is better reflected in overall survival (OS) and cancer specific survival (CSS). A randomized trial reported a higher rate of local recurrence for PN (6/268) compared to RN (1/273) [7], but the study did not separately examine whether the association between treatment and local recurrence was statistically significant. While there were studies supporting our results, a recent study by Gershman et al. revealed that, compared with PN, RN significantly associated with a marked reduction in local recurrence (HR = 0.19, 95% CI 0.11–0.35) [8]. Since the importance of nuclear grade in the prognosis of highly malignant renal tumors has been established [5, 20–22], assigning a greater complete resection range to patients with high nuclear grade can reduce the risk of postoperative recurrence, which supports clinical plausibility.

Surprisingly, the distant metastases-free curve showed statistical significance after OW of the data in the G3 subset (*P* < 0.05), but PN did not increase the risk of distant metastases after multivariate Cox regression modeling (*P* > 0.05). Given that the KM method is a nonparametric test and the Cox regression model is a semi-parametric test and can remove the impact of confounding factors, we believed that the results of the multivariate Cox regression model were more reliable.

Our results on the relationship between PN and distant metastases were different from those of previous studies [7, 8, 23–25]. We observed a significant association between RN and distant metastases in the G4 subset (HR = 0.094), with the five-year distant metastases-free survival rate increasing by 33.1%. In previous studies, which compared PN and RN for the treatment of T1 RCC, the number of patients with G4 RCC tended to be much smaller than that of the low grades. For example, the number of patients with G4 RCC treated with PN in the EORTC 30904 trial [7], as well as the studies of Gershman et al. [8], Simone et al. [22], Minervini et al. [23], and Antonelli et al. [24] were 1/268, 10/1175, 5/434, 1/332, and 13/1266 (G4/all grade), respectively. If the results are not analyzed separately, the substantial sample size of low nuclear grade groups may obscure the accurate results of the G4 group. Furthermore, in the RCC disease-free survival (DFS) model established by Correa, G1–G3 subsets associated with a significantly reduced risk of disease progression compared with the G4 subset (AF = 0.47, 95% CI 0.36, 0.62) [26]. This implied that G4 ccRCC was markedly more malignant than other nuclear grades, potentially impacting the efficacy of the operation. Therefore, we contend that, particularly for G4 patients, our study's results, which analyzed the G4 subset separately, are more reliable than previous studies with limited sample sizes for the G4 group. Finally, similar to local recurrence, assigning a wider resection range to G4 patients can reduce the risk of postoperative distant metastases also supports clinical plausibility.

No significant difference in ACM between PN and RN was observed, which is consistent with the results of previous studies [7, 8, 19, 22, 23]. Although some studies have reported an association between PN and the reduced risk of ACM [10, 18], this contradiction may be explained by the fact that RN associated with a lower postoperative eGFR [7, 8, 17, 18], and worsening renal function can increase in the incidence of complications, such as cardiovascular events [27], which can affect ACM. Of course, this may also be due to selection bias. Surprisingly, we did not observe a difference in ACM in the G4 subset, which had a large difference in the distant metastases-free survival rate. This was likely due to the short follow-up time (median follow-up time, 34 [IQR 26, 54] mo).

As for the laparoscopic and open groups. After applying the OW to balance baseline features, within the G4 subset, there was no significant correlation between the surgical approach (open vs. laparoscopic) and local recurrence (HR = 0.902, *p* = 1.108), distant metastasis (HR = 0.716, *p* = 0.570), or overall survival (HR = 0.580, *p* = 0.380). Similar results were observed in the other three nuclear grade subsets. This aligns with previous findings indicating that the outcomes of laparoscopic surgery are comparable to those of open surgery [28, 29].

Our study enrolled patients with pT1-stage ccRCC. If we wanted to extend these findings to patients with cT1-stage ccRCC, then we must consider that cT1 can be upstaged to pT3-4 compared with pT1. Shah reported that in patients upstaged to T3a from T1, PN was associated with a shorter recurrence-free survival compared to RN (HR = 2.04, 95% CI 1.12–3.68, *p* = 0.019) [30]. Thus, we provided a lower limit for the increased risk of recurrence associated with PN compared to RN for the cT1 stage.

There are several limitations in this study. Firstly, this was not a randomized trial, although we used OW to control the confounding factors brought about by non-randomness. However, PS method could not adjust for the patient features that were not measured and included in the PS model, so the possibility of residual confounding exists [14]. Secondly, our follow-up time was short (median follow-up time, 49 [IQR 26, 82] mo), especially in the G4 subset, which may have affected the estimation of the five-year rates. Thirdly, we conducted a multicenter study to expand the sample size, and a larger G4 ccRCC sample size was obtained compared with the other studies mentioned in the discussion [7, 8, 22–24]. However, the sample size of the G4 subset was still small. Then, owing to the belated adoption of robot-assisted surgery in the participating institutions, the count of patients undergoing robot-assisted PN/RN was limited. Additionally, patients in the participating institutions who had undergone robot-assisted surgery lacked an adequate follow-up period, prompting the exclusion of robot-assisted surgery as a covariate in our study. Finally, the cause of death was not available for some patients. Thus, to ensure the preciseness of the study, we used ACM instead of cancer-specific mortality and other-cause mortality as the outcome of the study.

Despite the above limitations, to our knowledge, this is the first study to compare the oncologic outcomes of PN and RN in four nuclear grade subsets. In comparison to previous studies with significant variations in nuclear grade sample sizes, our subset analysis enabled a more reliable comparison of the efficacy of RN and PN within high nuclear grade groups. Our study, for the first time, revealed that the risk of local ipsilateral recurrence and distant metastases is higher with PN than RN in the high nuclear grade (especially G4) group. Therefore, it is of great clinical significance to reassess the oncological equivalence of PN and RN in different nuclear grades. We will explore methods to enhance the preoperative determination of nuclear grade accuracy, aiming to offer more tailored surgical plans for patients with high nuclear grades.

## Conclusions

### Implications and key lessons learnt

Our results indicated that, in comparison to PN, RN was associated with a decreased risk of local ipsilateral recurrence in the G2, G3, and G4 subsets, a lowered risk of distant metastases in the G4 subset, and no significant difference in ACM among patients with pT1-ccRCC. Different from previous studies, our findings substantiate that opting for RN, as opposed to PN, is more advantageous for local recurrence-free survival and distant metastases-free survival in patients with high nuclear grade (especially G4) pT1-ccRCC. We recommend placing a heightened emphasis on enhancing preoperative nuclear grade assessment, as it can significantly influence the choice of surgical plan.

### Limitations

The main limitation of this study comes from the non-random design. An additional randomized controlled trial with a larger sample size of high nuclear grade ccRCC is warranted, as the need for high-quality prospective data remains.

### Supplementary Information

**Additional file1:** **Supplementary Table 1.** Comparisons of patient features by the type of nephrectomy in the pseudo G1 cohort after OW. **Supplementary Table 2.** Comparisons of patient features by the type of nephrectomy in the pseudo G2 cohort after OW. **Supplementary Table 3.** Comparisons of patient features by the type of nephrectomy in the pseudo G3 cohort after OW. **Supplementary Table 4.** Comparisons of patient features by the type of nephrectomy in the pseudo G4 cohort after OW.

## Data Availability

The datasets used and analysed during the current study are available from the corresponding author on reasonable request.
