# Supplementary material for: Patients with high nuclear grade pT1-ccRCC are more suitable for radical nephrectomy than partial nephrectomy: a multicenter retrospective study using propensity score
Source: World J Surg Oncol. 2024 Jan 23;22:24. doi: 10.1186/s12957-024-03302-y (PMC10804783; doi:10.1186/s12957-024-03302-y)
Supplement: Supplementary file 1 — Additional file1: Supplementary Table 1. Comparisons of patient features by the type of nephrectomy in the pseudo G1 cohort after OW. Supplementary Table 2. Comparisons of patient features by the type of nephrectomy in the pseudo G2 cohort after OW. Supplementary Table 3. Comparisons of patient features by the type of nephrectomy in the pseudo G3 cohort after OW. Supplementary Table 4. Comparisons of patient features by the type of nephrectomy in the pseudo G4 cohort after OW. [file 12957_2024_3302_MOESM1_ESM.docx]

**Supplementary material**

Supplementary Table 1: Comparisons of patient features by the type of nephrectomy in the pseudo G1 cohort after OW

Supplementary Table 2: Comparisons of patient features by the type of nephrectomy in the pseudo G2 cohort after OW

Supplementary Table 3: Comparisons of patient features by the type of nephrectomy in the pseudo G3 cohort after OW

Supplementary Table 4: Comparisons of patient features by the type of nephrectomy in the pseudo G4 cohort after OW

**Supplementary Table 1: Comparisons of patient features by the type of nephrectomy in the pseudo G1 cohort after OW.**

| **Feature** | **PN(N=76)** | **RN(N=88)** | **p value** |
| --- | --- | --- | --- |
| **Sex** |  |  |  |
| **Male** | 46 (61) | 55 (64) | 0.738 |
| **Female** | 30 (39) | 32 (36) |  |
| **Age(years)** | 53 (46-61) | 51 (44-59) | 0.104 |
| **Side** |  |  |  |
| **Left** | 35 (46) | 44 (50) | 0.616 |
| **Right** | 41 (54) | 44 (50) |  |
| **Tumor size(cm)** | 3.8 (3.0-4.2) | 3.6 (3.0-5.0) | 0.236 |
| **cT Stage** |  |  |  |
| **1a** | 47 (63) | 52 (60) | 0.751 |
| **1b** | 28 (37) | 35 (40) |  |
| **pT Stage** |  |  |  |
| **1a** | 47 (63) | 52 (60) | 0.751 |
| **1b** | 28 (37) | 35 (40) |  |
| **Surgical approach** |  |  |  |
| **Open** | 8 (11) | 10 (11) | 0.946 |
| **Laparoscopic** | 67 (89) | 77 (89) |  |
| **Lumbago** |  |  |  |
| **No** | 65 (87) | 74 (84) | 0.712 |
| **Yes** | 10 (13) | 14 (16) |  |
| **Hematuresis** |  |  |  |
| **No** | 73 (97) | 84 (95) | 0.680 |
| **Yes** | 2 (3) | 4 (5) |  |
| **Cardiovascular disease** |  |  |  |
| **No** | 54 (71) | 65 (75) | 0.615 |
| **Yes** | 22 (39) | 22 (25) |  |
| **Smoking status** |  |  |  |
| **Never** | 55 (72) | 64 (73) | 0.984 |
| **<10 years** | 5 (7) | 7 (8) |  |
| **10-19 years** | 8 (11) | 8 (9) |  |
| **20-29 years** | 4 (5) | 6 (7) |  |
| **≥30 years** | 3 (4) | 3 (3) |  |
| **Preoperative eGFR** | 74.3 (58.4-89.2) | 78.6 (65.0-93.2) | <0.05 |
| **Preoperative eGFR status** |  |  |  |
| **≥90** | 18 (24) | 26 (30) | 0.515 |
| **60-90** | 37 (49) | 44 (50) |  |
| **30-60** | 19 (25) | 16 (18) |  |
| **15-30** | 1 (1) | 0 (0) |  |
| **≤15** | 1 (1) | 0 (0) |  |
| **aCCI score** | 2 (2.0-2.0) | 2 (2.0-2.0) | 0.246 |
| **ECOG performance status** |  |  |  |
| **0** | 27 (36) | 37 (42) | 0.472 |
| **1** | 40 (52) | 44 (51) |  |
| **2** | 9 (12) | 6 (7) |  |
| **3** | 0 (0) | 0 (0) |  |
| **BMI** | 23.5 (21.4-26.2) | 23.5 (21.7-25.7) | 0.354 |
| **Radiographic evidence of hemorrhage** |  |  |  |
| **No** | 70 (92) | 81 (92) | 0.924 |
| **Yes** | 6 (8) | 7 (8) |  |
| **Radiographic evidence of cystic** |  |  |  |
| **No** | 67 (89) | 77 (89) | 0.924 |
| **Yes** | 8 (11) | 10 (11) |  |
| **Radiographic evidence of calcification** |  |  |  |
| **No** | 72 (96) | 81 (93) | 0.408 |
| **Yes** | 3 (4) | 6 (7) |  |
| **Radiographic evidence of necrosis** |  |  |  |
| **No** | 75 (100) | 87 (99) | 0.375 |
| **Yes** | 0 (0) | 1 (1) |  |
| **aCCI = age-adjusted Charlson Comorbidity Index; BMI = body mass index; ECOG = Eastern Cooperative Oncology Group; eGFR = estimated glomerular filtration rate; OW=overlap weighting; IQR = interquartile range; PN = partial nephrectomy; RN = radical nephrectomy.**  **Numbers represent median (IQR) or N (%).** | | | |

**Supplementary Table 2: Comparisons of patient features by the type of nephrectomy in the pseudo G2 cohort after OW.**

| **Feature** | **PN(N=177)** | **RN(N=163)** | **p value** |
| --- | --- | --- | --- |
| **Sex** |  |  |  |
| **Male** | 115 (65) | 106 (66) | 0.920 |
| **Female** | 62 (35) | 56 (34) |  |
| **Age(years)** | 54 (46-62) | 55 (47-63) | 0.366 |
| **Side** |  |  |  |
| **Left** | 85 (48) | 73 (45) | 0.582 |
| **Right** | 92 (52) | 90 (55) |  |
| **Tumor size(cm)** | 4.0 (3.1-4.7) | 3.9 (3.0-4.5) | <0.05 |
| **cT Stage** |  |  |  |
| **1a** | 96 (54) | 101 (63) | 0.138 |
| **1b** | 81 (46) | 61 (37) |  |
| **pT Stage** |  |  |  |
| **1a** | 96 (54) | 101 (63) | 0.138 |
| **1b** | 81 (46) | 61 (37) |  |
| **Surgical approach** |  |  |  |
| **Open** | 18 (10) | 11 (7) | 0.221 |
| **Laparoscopic** | 159 (90) | 152 (93) |  |
| **Lumbago** |  |  |  |
| **No** | 158 (90) | 143 (88) | 0.645 |
| **Yes** | 18 (10) | 19 (12) |  |
| **Hematuresis** |  |  |  |
| **No** | 166 (94) | 152 (94) | 0.884 |
| **Yes** | 11 (7) | 10 (7) |  |
| **Cardiovascular disease** |  |  |  |
| **No** | 131 (75) | 117 (72) | 0.584 |
| **Yes** | 45 (25) | 46 (28) |  |
| **Smoking status** |  |  |  |
| **Never** | 133 (75) | 120 (74) | 0.893 |
| **<10 years** | 10 (6) | 9 (6) |  |
| **10-19 years** | 18 (10) | 15 (9) |  |
| **20-29 years** | 11 (6) | 10 (6) |  |
| **≥30 years** | 5 (3) | 8 (5) |  |
| **Preoperative eGFR** | 73.6 (60.1-90.0) | 74.8 (59.4-89.4) | 0.172 |
| **Preoperative eGFR status** |  |  |  |
| **≥90** | 44 (25) | 39 (24) | 0.826 |
| **60-90** | 89 (50) | 81 (50) |  |
| **30-60** | 41 (23) | 37 (23) |  |
| **15-30** | 2 (1) | 4 (2) |  |
| **≤15** | 1 (1) | 1 (1) |  |
| **aCCI score** | 2.0 (2.0-2.0) | 2.0 (2.0-2.0) | 0.904 |
| **ECOG performance status** |  |  |  |
| **0** | 94 (53) | 80 (49) | 0.843 |
| **1** | 69 (39) | 71 (44) |  |
| **2** | 14 (8) | 11 (7) |  |
| **3** | 1 (1) | 1 (1) |  |
| **BMI** | 24.2 (22.1-26.6) | 23.7 (21.7-26.1) | 0.699 |
| **Radiographic evidence of hemorrhage** |  |  |  |
| **No** | 170 (97) | 155 (95) | 0.640 |
| **Yes** | 6 (3) | 8 (5) |  |
| **Radiographic evidence of cystic** |  |  |  |
| **No** | 165 (93) | 152 (94) | 0.930 |
| **Yes** | 12 (7) | 10 (6) |  |
| **Radiographic evidence of calcification** |  |  |  |
| **No** | 173 (98) | 161 (99) | 0.456 |
| **Yes** | 4 (2) | 2 (1) |  |
| **Radiographic evidence of necrosis** |  |  |  |
| **No** | 174 (98) | 160 (99) | 0.873 |
| **Yes** | 3 (2) | 2 (1) |  |
| **aCCI = age-adjusted Charlson Comorbidity Index; BMI = body mass index; ECOG = Eastern Cooperative Oncology Group; eGFR = estimated glomerular filtration rate; OW=overlap weighting; IQR = interquartile range; PN = partial nephrectomy; RN = radical nephrectomy.**  **Numbers represent median (IQR) or N (%).** | | | |

**Supplementary Table 3: Comparisons of patient features by the type of nephrectomy in the pseudo G3 cohort after OW.**

| **Feature** | **PN(N=55)** | **RN(N=54)** | **p value** |
| --- | --- | --- | --- |
| **Sex** |  |  |  |
| **Male** | 40 (74) | 37 (69) | 0.557 |
| **Female** | 14 (26) | 17 (31) |  |
| **Age(years)** | 54 (47-63) | 56 (47-62) | 0.777 |
| **Side** |  |  |  |
| **Left** | 28 (51) | 28 (53) | 0.874 |
| **Right** | 27 (49) | 25 (47) |  |
| **Tumor size(cm)** | 4.0 (3.0-5.0) | 4.5 (3.5-5.0) | <0.05 |
| **cT Stage** |  |  |  |
| **1a** | 34 (63) | 22 (42) | <0.05 |
| **1b** | 20 (37) | 31 (58) |  |
| **pT Stage** |  |  |  |
| **1a** | 34 (63) | 22 (42) | <0.05 |
| **1b** | 20 (37) | 31 (58) |  |
| **Surgical approach** |  |  |  |
| **Open** | 3 (5) | 9 (18) | <0.05 |
| **Laparoscopic** | 52 (95) | 44 (82) |  |
| **Lumbago** |  |  |  |
| **No** | 46 (85) | 50 (93) | 0.181 |
| **Yes** | 8 (15) | 4 (7) |  |
| **Hematuresis** |  |  |  |
| **No** | 51 (93) | 51 (95) | 0.592 |
| **Yes** | 4 (7) | 2 (5) |  |
| **Cardiovascular disease** |  |  |  |
| **No** | 38 (70) | 40 (74) | 0.627 |
| **Yes** | 16 (30) | 14 (26) |  |
| **Smoking status** |  |  |  |
| **Never** | 41 (75) | 40 (74) | 0.769 |
| **<10 years** | 3 (5) | 2 (4) |  |
| **10-19 years** | 6 (11) | 8 (15) |  |
| **20-29 years** | 4 (7) | 3 (6) |  |
| **≥30 years** | 2 (4) | 0 (0) |  |
| **Preoperative eGFR** | 78.9 (67.3-92.4) | 76.2 (60.6-95.2) | 0.533 |
| **Preoperative eGFR status** |  |  |  |
| **≥90** | 15 (27) | 18 (33) | 0.329 |
| **60-90** | 32 (58) | 23 (43) |  |
| **30-60** | 8 (15) | 11 (20) |  |
| **15-30** | 0 (0) | 1 (2) |  |
| **≤15** | 0 (0) | 0 (0) |  |
| **aCCI score** | 2.0 (2.0-2.0) | 2.0 (2.0-2.0) | 0.800 |
| **ECOG performance status** |  |  |  |
| **0** | 35 (64) | 34 (63) | 0.273 |
| **1** | 19 (35) | 15 (28) |  |
| **2** | 1 (2) | 4 (7) |  |
| **3** | 0 (0) | 0 (0) |  |
| **BMI** | 23.2 (21.9-25.7) | 24.0 (21.6-25.4) | 0.433 |
| **Radiographic evidence of hemorrhage** |  |  |  |
| **No** | 53 (97) | 53 (100) | 0.194 |
| **Yes** | 1 (3) | 0 (0) |  |
| **Radiographic evidence of cystic** |  |  |  |
| **No** | 53 (96) | 51 (95) | 0.898 |
| **Yes** | 2 (4) | 2 (5) |  |
| **Radiographic evidence of calcification** |  |  |  |
| **No** | 54 (98) | 53 (100) | 0.223 |
| **Yes** | 1 (2) | 0 (0) |  |
| **Radiographic evidence of necrosis** |  |  |  |
| **No** | 54 (98) | 53 (98) | 0.897 |
| **Yes** | 1 (2) | 1 (2) |  |
| **aCCI = age-adjusted Charlson Comorbidity Index; BMI = body mass index; ECOG = Eastern Cooperative Oncology Group; eGFR = estimated glomerular filtration rate; OW=overlap weighting; IQR = interquartile range; PN = partial nephrectomy; RN = radical nephrectomy.**  **Numbers represent median (IQR) or N (%).** | | | |

**Supplementary Table 4: Comparisons of patient features by the type of nephrectomy in the pseudo G4 cohort after OW.**

| **Feature** | **PN(N=10)** | **RN(N=14)** | **p value** |
| --- | --- | --- | --- |
| **Sex** |  |  |  |
| **Male** | 8 (80) | 11 (79) | 0.862 |
| **Female** | 2 (20) | 3 (21) |  |
| **Age(years)** | 62 (54-63) | 59 (50-71) | 0.702 |
| **Side** |  |  |  |
| **Left** | 3 (33) | 6 (46) | 0.623 |
| **Right** | 6 (67) | 7 (54) |  |
| **Tumor size(cm)** | 4.7 (4.0-5.7) | 4.5 (4.0-5.0) | 0.998 |
| **cT Stage** |  |  |  |
| **1a** | 3 (30) | 4 (31) | 0.810 |
| **1b** | 7 (70) | 9 (69) |  |
| **pT Stage** |  |  |  |
| **1a** | 3 (30) | 4 (31) | 0.810 |
| **1b** | 7 (70) | 9 (69) |  |
| **Surgical approach** |  |  |  |
| **Open** | 2 (20) | 1 (8) | 0.502 |
| **Laparoscopic** | 8 (80) | 12 (92) |  |
| **Lumbago** |  |  |  |
| **No** | 8 (80) | 12 (86) | 0.759 |
| **Yes** | 2 (20) | 2 (14) |  |
| **Hematuresis** |  |  |  |
| **No** | 9 (90) | 12 (92) | 0.869 |
| **Yes** | 1 (10) | 1 (8) |  |
| **Cardiovascular disease** |  |  |  |
| **No** | 8 (80) | 10 (77) | 0.651 |
| **Yes** | 2 (20) | 3 (23) |  |
| **Smoking status** |  |  |  |
| **Never** | 7 (78) | 11 (84) | 0.757 |
| **<10 years** | 0 (0) | 0 (0) |  |
| **10-19 years** | 0 (0) | 1 (8) |  |
| **20-29 years** | 0 (0) | 0 (0) |  |
| **≥30 years** | 2 (22) | 1 (8) |  |
| **Preoperative eGFR** | 73.6 (60.6-85.6) | 71.8 (54.5-83.1) | 0.958 |
| **Preoperative eGFR status** |  |  |  |
| **≥90** | 1 (11) | 2 (15) | 0.583 |
| **60-90** | 6 (67) | 6 (46) |  |
| **30-60** | 2 (22) | 5 (38) |  |
| **15-30** | 0 (0) | 0 (0) |  |
| **≤15** | 0 (0) | 0 (0) |  |
| **aCCI score** | 2.0 (2.0-2.0) | 2.0 (2.0-2.0) | 0.103 |
| **ECOG performance status** |  |  |  |
| **0** | 3 (30) | 7 (54) | 0.273 |
| **1** | 6 (60) | 4 (31) |  |
| **2** | 1 (10) | 2 (15) |  |
| **3** | 0 (0) | 0 (0) |  |
| **BMI** | 21.7 (20.4-24.2) | 21.9 (20.6-25.1) | 0.268 |
| **Radiographic evidence of hemorrhage** |  |  |  |
| **No** | 9 (100) | 13 (100) | 0.357 |
| **Yes** | 0 (0) | 0 (0) |  |
| **Radiographic evidence of cystic** |  |  |  |
| **No** | 8 (80) | 12 (92) | 0.515 |
| **Yes** | 2 (20) | 1 (8) |  |
| **Radiographic evidence of calcification** |  |  |  |
| **No** | 10 (100) | 13 (100) | NA |
| **Yes** | 0 (0) | 0 (0) |  |
| **Radiographic evidence of necrosis** |  |  |  |
| **No** | 10 (100) | 13 (100) | 0.960 |
| **Yes** | 0 (0) | 0 (0) |  |
| **aCCI = age-adjusted Charlson Comorbidity Index; BMI = body mass index; ECOG = Eastern Cooperative Oncology Group; eGFR = estimated glomerular filtration rate; NA= not available; OW=overlap weighting; IQR = interquartile range; PN = partial nephrectomy; RN = radical nephrectomy.**  **Numbers represent median (IQR) or N (%).** | | | |
